# DUSP6 deletion protects mice and reduces disease severity in autoimmune arthritis

**DOI:** 10.1016/j.isci.2024.110158

**Published:** 2024-05-31

**Authors:** Teresina Laragione, Carolyn Harris, Natasha Rice, Percio S. Gulko

**Affiliations:** 1 Division of Rheumatology, Department of Medicine, Icahn School of Medicine at Mount Sinai, New York, NY 10029, USA

**Keywords:** Health sciences, Biological sciences, Physiology, Molecular biology, Immunology

## Abstract

Receptor tyrosine kinases (RTKs) have an important role in arthritis severity and in models of rheumatoid arthritis (RA), but their regulation is not fully understood. The dual specificity phosphatase 6 (DUSP6) has been implicated in the regulation of RTK signaling, but never in the context of arthritis and autoimmunity. We used the KRN serum-induced arthritis (KSIA) model of RA and showed that DUSP6^−/−^ mice were protected and had a 50% lower maximum arthritis score (*p* = 0.006) and reduced joint damage than C57BL/6 DUSP6+/+ controls. Serum levels of interleukin (IL) 10 were significantly increased (>2-fold), and IL6 decreased in DUSP6^−/−^ mice. DUSP6^−/−^ mice had increased numbers of IL10+ cells including Tr1 regulatory cells (*p* < 0.01). Introduction of the IL10^−/−^ into DUSP6^−/−^ (double knockout [KO]) reversed the DUSP6^−/−^ protection. In conclusion, this study reports a pro-arthritic role for DUSP6. This discovery has the potential to generate a previously unknown target for therapies for RA and inflammatory diseases.

## Highlights

• DUSP6 is associated with increased disease severity and joint damage in autoimmune arthritis
• Mice with a DUSP6 deletion are significantly protected, preserving normal joints
• DUSP6 knockout mice exhibit increased serum levels of IL10 and IL10-producing cells but reduced IL6
• DUSP6 is a potential therapeutic target for autoimmune and inflammatory diseases such as RA

## Graphical abstract

**Figure:**
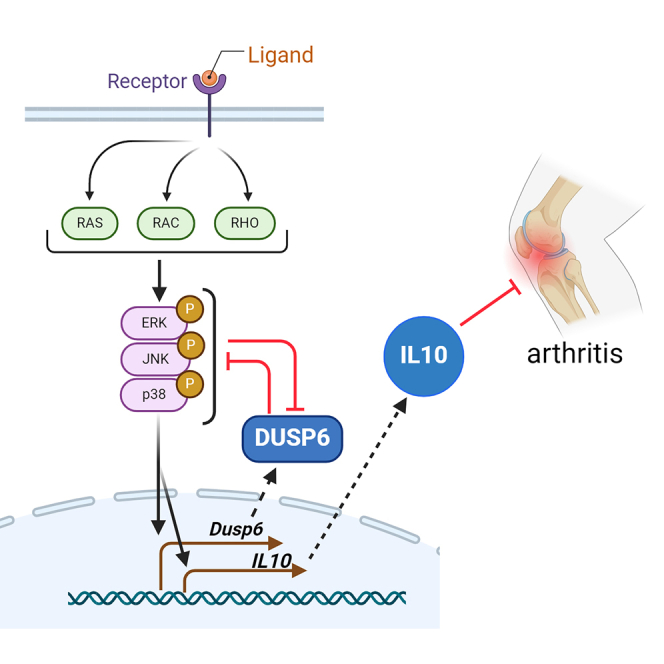

## Introduction

Disease remission remains uncommon in rheumatoid arthritis (RA), and new and better treatments are needed. In order to develop new treatments, a better understanding of disease pathogenesis is required, with the identification of new therapeutic targets. Our previous studies showed that the Huntingtin-interacting protein 1 (HIP1) contributes to the invasive properties of RA fibroblast-like synoviocytes (FLSs) and of arthritis severity.^1^ HIP1 mediates receptor tyrosine kinase (RTK) signaling, underscoring the importance of these receptors and the RTK signaling pathway to RA.^1^ Several proteins and pathways mediate RTK intracellular signaling, and the dual specificity phosphatase 6 (DUSP6) has a central role in part of these processes.^2^^,^^3^ DUSP6 de-phosphorylates tyrosine and threonine to inactivate Erk and potentially other mitogen-activated protein (MAP) kinase proteins, which in turn regulate DUSP6 expression in a negative feedback.^2^^,^^4^^,^^5^ DUSP6 is expressed by RA synovial tissue-infiltrating T cells, B cells, and macrophages.^6^ Therefore, we hypothesized that DUSP6 might have a role in the pathogenesis of RA and in rodent models of RA.

In the present study we report that the deletion of DUSP6 significantly protected mice in a model of RA induced with the KRN arthritogenic serum. The DUSP6 knockout (KO) mice preserve a normal joint histology without erosions. We identified differences in B and T cell populations in DUSP6 KO mice and reduced expression of pro-inflammatory cytokines. We also show that the protective effect correlates with increased levels of interleukin (IL) 10 and is dependent on the production of IL10. These studies report a key role for DUSP6 in a model of RA and suggest that it may be a potential target for treatment.

## Results

### DUSP6 KO mice are protected and developed milder arthritis

**Figure 1:**
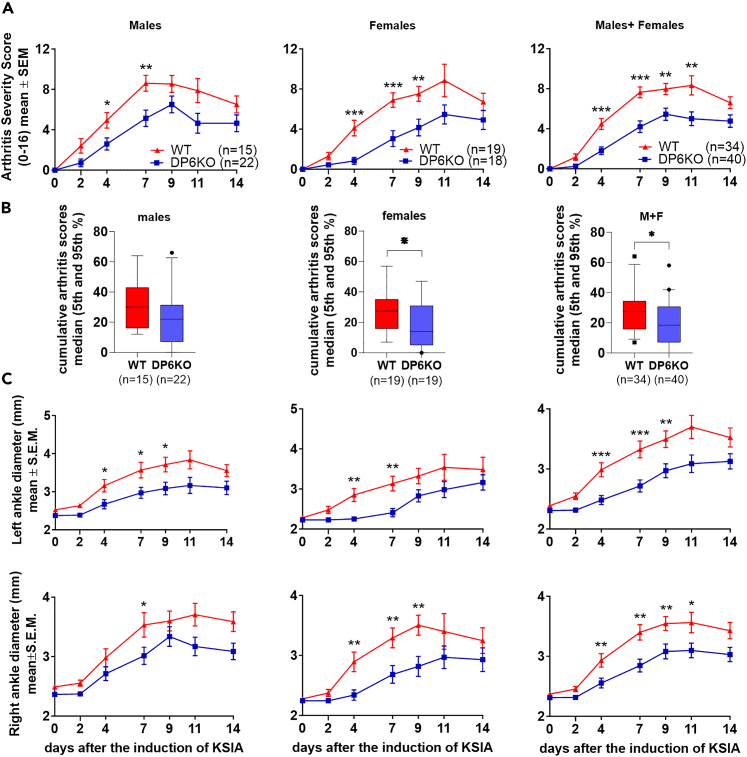
Male and female DUSP6 KO and WT arthritis severity scoring in KSIA (A) DUSP6 KO (DP6KO) males had significantly lower arthritis severity scored compared with C57BL/6 (WT). The difference was detected as early as day 4 (WT *n* = 15, DUSP6 KO *n* = 22, ∗*p* = 0.019; ∗∗*p* = 0.006). DUSP6 KO female mice also had significantly lower arthritis score (WT *n* = 19, DUSP6 KO *n* = 19, ∗∗∗*p* < 0.006; ∗p≤ = 0.05). Males and females were also analyzed together (males+females). (B) To analyze the area under the curves, the medians of the cumulative arthritis scores over time were compared. For combined males+females the median of the cumulative arthritis severity scores (DUSP6 KO median = 18.5; *n* = 40; WT median = 27.5; *n* = 34) was significantly different (∗*p* = 0.0115). The female group comparison (Fem DUSP6 KO median = 14; *n* = 19; Fem WT median = 27.5; *n* = 19) was also significantly different (∗*p* = 0.05). The median cumulative arthritis scores of males were lower in the DUSP6 KO mice compared with WT (male DUSP6 KO median = 22; *n* = 22; WT median = 30; *n* = 15) but did not reach statistical significance. (C) Ankle diameters were significantly smaller in male and female KO mice compared with WT at day 4, 7, and 9 (male WT *n* = 15, DUSP6 KO *n* = 23: female WT *n* = 19, DUSP6 KO *n* = 17; ∗*p* ≤ = 0.05, ∗∗*p* < 0.002; t test).

Male and female DUSP6 KO and wild-type (WT) mice were studied in KRN serum-induced arthritis (KSIA) for fourteen days. DUSP6 KO males were protected and had lower arthritis severity scores compared with WT mice (Figure 1A). The difference was significant as early as day 4 (Figure 1; WT *n* = 15, DUSP6 KO *n* = 22, ∗*p* = 0.019; ∗∗*p* = 0.006). DUSP6 KO female mice also had significantly lower arthritis score (Figure 1A; WT *n* = 19, DUSP6 KO *n* = 18, ∗∗∗*p* < 0.006; ∗*p* = 0.02). The analyses of the median cumulative arthritis scores over time, representing the area under the curve, also revealed significantly lower scores in the combined male + female group (*p* = 0.0115; Mann-Whitney test), and in the female DUSP6 KO group, compared with WT (*p* = 0.05; Figure 1B). The median cumulative arthritis scores were lower in the male KO mice compared with WT but did not reach statistical significance (Figure 1B). Ankle diameters were also smaller in male and female KO mice compared with WT (Figure 1C; ∗*p* < 0.02, ∗∗*p* < 0.002).

**Figure 2:**
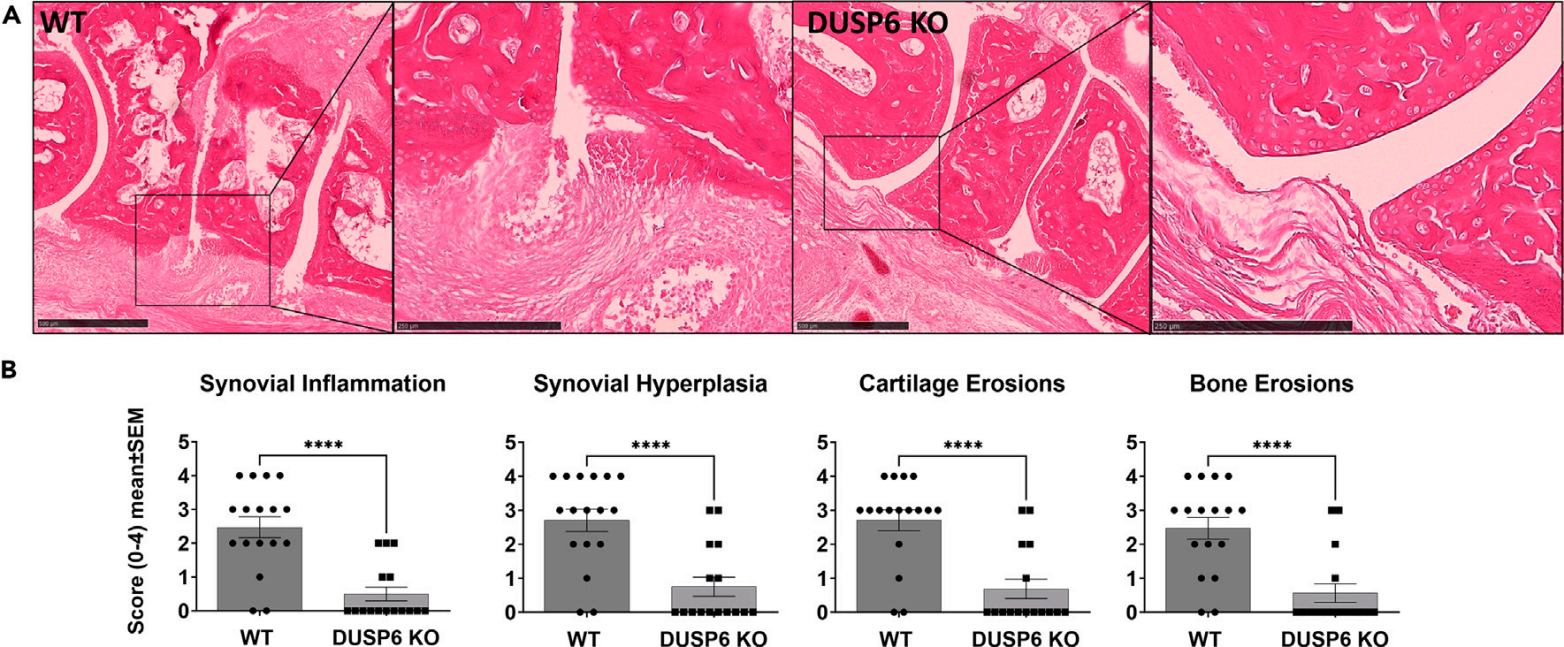
Ankle histology and histology scoring (A) C57BL/6 (WT; *n* = 17) ankle joints had pronounced synovial hyperplasia and erosive changes (ankles collected on day 9 of KSIA; size bar is 500 μm on main image and 250 μm in the magnified area), while DUSP6 KO (*n* = 16) preserved a normal joint architecture with no significant synovial hyperplasia or erosions. (B) Histology sections were scored as described, and DUSP6 KO mice had significantly lower scores for inflammation, synovial hyperplasia, and cartilage and bone erosions (t test, ∗∗∗∗*p* < 0.0001).

Histology analysis was done on ankles obtained at the end of the 14-day KSIA study. DUSP6 KO mice had a nearly normal architecture, with significantly lower scores for synovial hyperplasia, synovial inflammation, and cartilage and bone erosions, compared with WT mice (Figures 2A and 2B).

### Increased serum levels of IL10 and reduced levels of IL6 in DUSP6 KO mice

**Figure 3:**
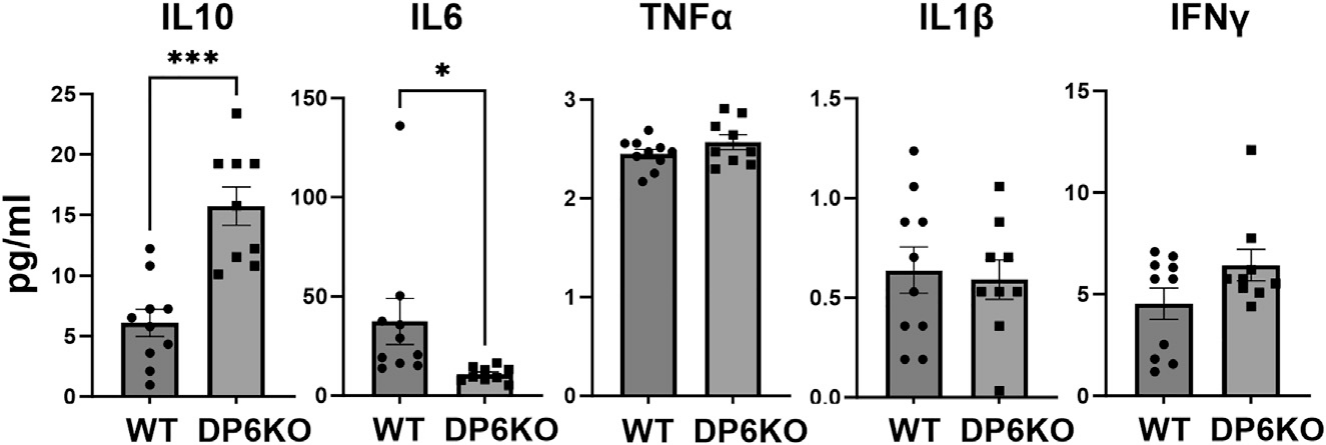
Serum levels of cytokines in DUSP6 KO and C57BL/6 (WT) with KSIA Serum levels of IL10 were significantly higher in DUSP6 KO (*n* = 9) versus WT mice (*n* = 10) (greater that 2-fold higher; ∗∗∗*p* = 0.0001). Serum levels of IL6 were significantly lower in the DUSP6 KO mice (less than 50%; ∗*p* = 0.03). Levels of IL1β and TNF-α were not significantly different between the two groups. Levels of IFNγ were higher in the KO mice, but that was not statistically significant.

Serum was collected from male mice at the peak of KSIA (day 9). Levels of IL10 were significantly increased by nearly 3-fold in DUSP6 KO serum compared with WT mice (Figure 3; ∗∗∗*p* = 0.0001). Serum levels of IL6 were significantly lower in DUSP6 KO mice at nearly 50% of the levels of WT mice (Figure 3; ∗*p* = 0.03). Levels of tumor necrosis factor alpha (TNF-α) and IL1β were very low and not significantly different between KO and WT (Figure 3). Levels of interferon (IFN) γ were not significantly different (Figure 3).

### DUSP6 KO mice have increased numbers of IL10+ cells

**Figure 4:**
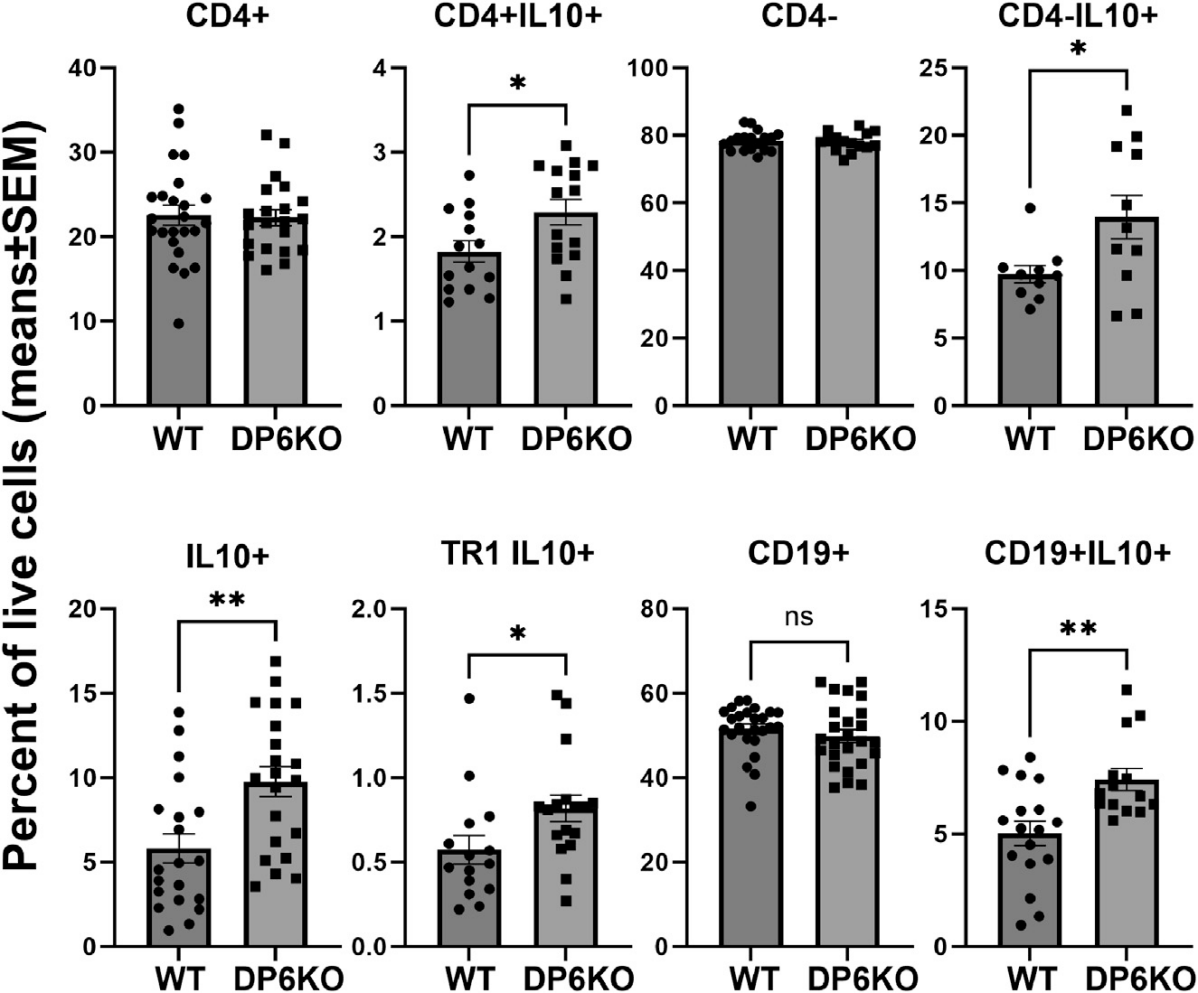
Flow cytometry analysis of male splenocytes in KSIA Numbers of total CD4^+^ T cells and CD19^+^ B cells did not differ between DUSP6 KO (*n* = 21) and WT (*n* = 23). However, IL10+ cells were increased in DUSP6 KO mice compared with C57BL/6 (WT). Both CD4+IL10+ and CD4-IL10+ cells were significantly increased in DUSP6 KO (DP6 KO) mice, compared with WT mice (∗*p* ≤ 0.04). IL10-producing Tr1 cells (CD4^+^CD45^−^CD49b+LAG3+IL10+) and CD19+IL10 + B cells were also increased in DUSP6 KO (∗*p* ≤ 0.04).

We next examined whether the deletion of DUSP6 could affect the numbers of T and B cell subsets implicated in arthritis and autoimmunity. Given the increased serum levels of IL10 in DUSP6 KO mice, we aimed to determine the potential cellular source of IL10. Flow cytometry analyses of male splenocytes obtained on day 9 (disease peak) following the induction of KSIA revealed increased total number of IL10+ cells in DUSP6 KO mice. That included an increased number of CD4+IL10+ (Figure 4; *p* = 0.03), CD4-IL10+ (Figure 4; *p* = 0.03), and Tr1 (CD4^+^CD45^−^CD49b+LAG3+IL10+) cells (Figure 4; *p* = 0.008). Numbers of CD19+IL10 + B cells were also increased in DUSP6 KO males (Figure 4; *p* = 0.007; see Figure S1 for gating). Numbers of Th17 cells, Tfh cells, total CD4+Foxp3+, CD4+Foxp3+IL10+ Tregs, and myeloid CD11b+Gr1+ were not significantly different between DUSP6 KO and WT (see Figure S2).

### The introduction of the IL10 KO reverses the arthritis protection seen in DUSP6 KO

Following the observation of increased serum levels of IL10, as well as increased frequency of IL10+ cells in the DUSP6 KO mice, we hypothesized that IL10 was mediating the arthritis-protective effect observed in DUSP6 KO mice. To test that hypothesis, we generated IL10 and DUSP6 double KO mice.

**Figure 5:**
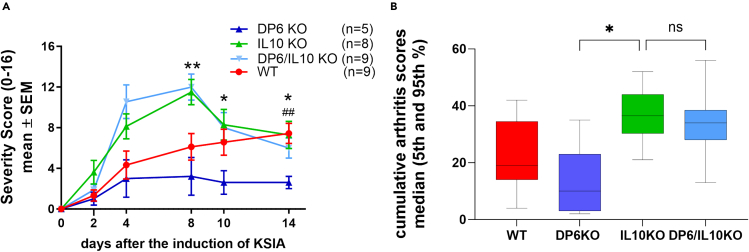
KSIA studies in IL10/DUSP6 double KO mice (A) DUSP6 KO (DP6KO; dark blue line; *n* = 5) males had significantly lower arthritis severity scores compared with C57BL/6 (WT; red line; *n* = 9; ##*p* = 0.0039, t test). IL10 KO mice (green line; *n* = 8) were not protected and developed arthritis more severe than the WT mice. IL10 KO introduced into the DUSP6 KO completely reversed the protection conferred by the DUSP6 KO, and double KO mice (light blue line; *n* = 9) developed disease similar to the IL10 KO, and significantly more severe than the WT and DUSP6 KO (∗*p* = 0.02; ∗∗*p* = 0.001; t test). (B) Cumulative arthritis scores (area under the curve) from the same experiment shown in (A) showing lower median scores in DUSP6 KO (dark blue) compared with C57BL/6 (WT; red), IL10 KO (green), and DUSP6/IL10 double KO mice (light blue) (∗*p* = 0.0132, Kruskal-Wallis test).

While DUSP6 KO mice were again protected in the KSIA model, the IL10 KO and the double KO mice developed severe disease with arthritis scores higher than those of the C57BL/6 WT mice (Figures 5A and 5B). These observations confirmed that the DUSP6 KO arthritis protection is dependent on the production of IL10.

### Transcriptomic analysis of spleens from WT and DUSP6 KO mice with KSIA revealed differences in Erk pathway and cytokine regulation and secretion

We used RNA sequencing to analyze gene expression in the spleens from WT and DUSP6 KO mice at day 9 after KSIA. These studies aimed at characterizing pathways and cellular processes significantly affected by the presence or absence of DUSP6.

**Figure 6:**
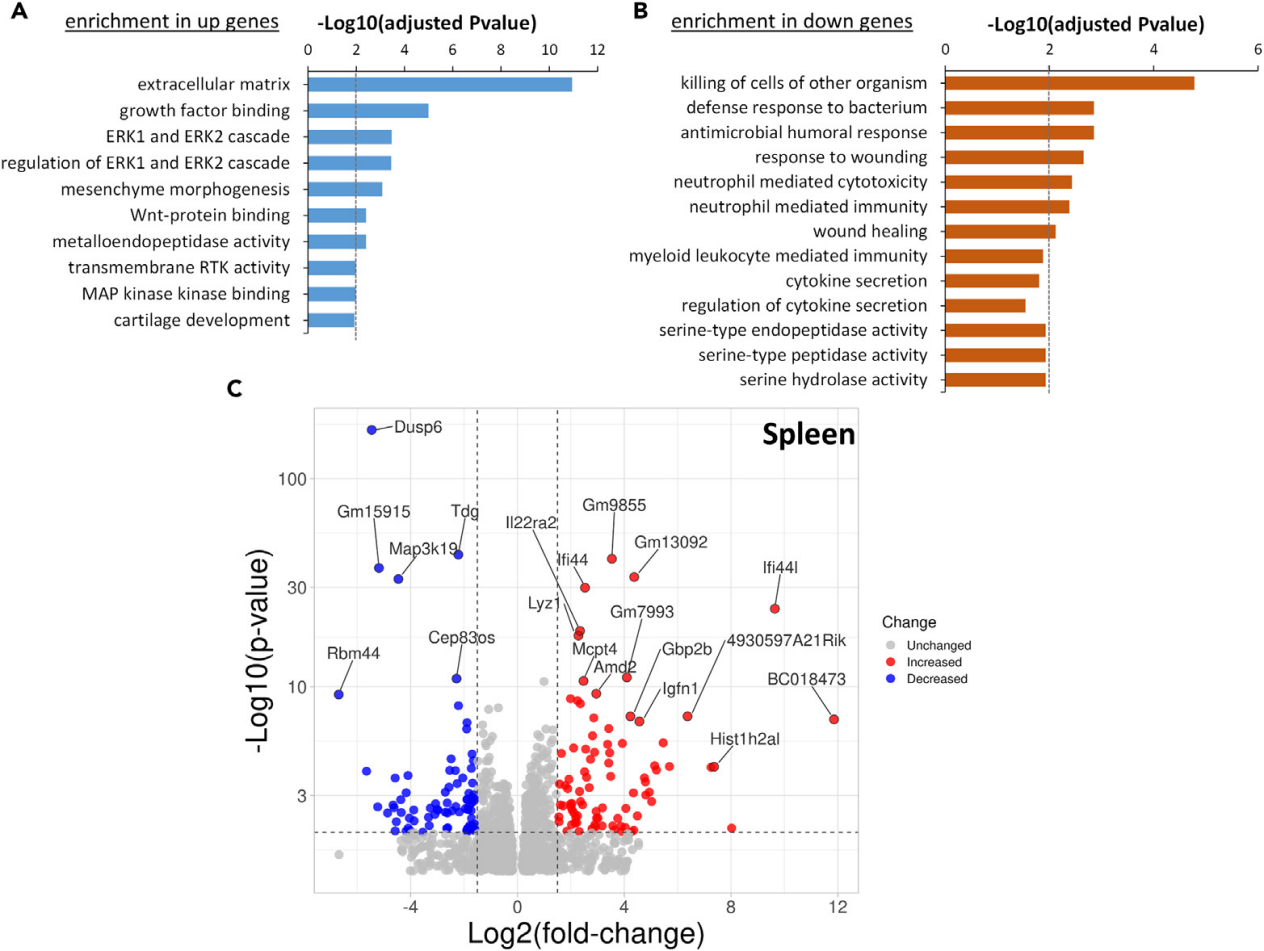
RNA sequencing and analyses of spleens from DUSP6 KO and C57BL/6 (WT) mice with KSIA (A) GO pathways and processes enriched among the genes expressed in increased levels in DUSP6 KO mice, compared with WT mice. (B) GO pathways and process enriched among the genes expressed in lower levels in DUSP6 KO mice. (C) Volcano plot showing the differentially expressed genes (red = increased expression in DUSP6 KO; blue = decreased expression in DUSP6 KO; gray = unchanged).

There were 657 genes expressed in increased levels and 705 expressed in decreased levels in spleens of DUSP6 KO mice, compared with WT. Among the genes expressed in increased levels DUSP6 KO spleens, there was a significant enrichment for Gene Ontology (GO) pathways and processes involved in “regulation of ERK1 and ERK2 cascade” and “negative regulation of ERK1 and ERK2 cascade” (Figure 6A), which is a pathway known to be regulated by DUSP6. “Cartilage development,” “regulation of cellular response to growth factor stimulus,” and “transmembrane receptor tyrosine kinase activity” were also among the processes enriched in DUSP6 KO (Figure 6A; Table S1).

Among the genes expressed in decreased levels in spleens from DUSP6 KO mice, there was a significant enrichment for GO pathways and processes involved in “regulation of cytokine secretion,” “cytokine secretion,” “serine-type endopeptidase activity,” “neutrophil-mediated toxicity,” “neutrophil-mediated immunity,” and others (Figure 6B; Table S2).

Genes with the most significantly increased expression in the spleens of DUSP6 KO mice included Ifi44l, IL22ra2, Igfn1, Spry1, Spy2, and Spry4 (Figure 6C, Tables 1 and S3). The genes with the most significantly decreased expression in the spleens of DUSP6 KO mice included DUSP6 (as expected), DUSP11, Map3k19, Tdg, Vcan, Lum, and Utp14b (Figure 6C, Tables 1 and S4).

**Table 1:** Selected list of the most significantly differentially expressed genes (DEGs) in DUSP6 KO spleens compared with WT controls

Gene name	Gene description	log2FoldChange	*p* value	adjusted *p* value^a^
**Lowest expression in DUSP6 KO spleens**				

Dusp6	dual specificity phosphatase 6	−5.455637762	4.21E-172	8.31E-168
Tdg	thymine DNA glycosylase	−2.210974592	7.65E-44	7.55E-40
Gm15915	predicted gene 15915	−5.184162876	6.20E-38	3.06E-34
Map3k19	mitogen-activated protein kinase kinase kinase 19	−4.457313704	1.20E-33	3.95E-30
Cep83os	centrosomal protein 83, opposite strand	−2.279335737	1.14E-11	1.87E-08
Prss34	protease, serine 34	−2.208480251	7.72E-09	7.61E-06
Lta4h	leukotriene A4 hydrolase	−0.712882833	1.19E-08	1.12E-05
Igkv14-126	immunoglobulin kappa variable 14-126	−1.074613836	1.62E-08	1.45E-05
Vcan	versican	−1.886980899	1.87E-07	0.000132086
Fmod	fibromodulin	−1.305573056	2.73E-07	0.000185929
Utp14b	UTP14B small subunit processome component	−1.28150536	1.10E-06	0.000637502
Hspb1	heat shock protein 1	−1.023999559	3.11E-06	0.001534878
Lum	lumican	−2.482552511	3.13E-05	0.009803572
Serpina3b	serine (or cysteine) peptidase inhibitor, clade A, member 3B	−1.649480753	3.84E-05	0.011650612

**Highest expression in DUSP6 KO spleens**				

Ifi44	interferon-induced protein 44	2.531444893	1.22E-30	3.44E-27
Ifi44l	interferon-induced protein 44 like	9.64041653	1.93E-24	4.76E-21
Il22ra2	interleukin-22 receptor, alpha 2	2.3451977	2.84E-19	6.21E-16
Lyz1	lysozyme 1	2.285229623	2.46E-18	4.86E-15
Mcpt4	mast cell protease 4	2.475596145	2.19E-11	3.33E-08
Gbp2b	guanylate binding protein 2b	4.232183645	6.24E-08	5.13E-05
Igfn1	immunoglobulin-like and fibronectin type III domain 1	4.570498524	1.53E-07	0.000112111
Spry1	sprouty RTK signaling antagonist 1	1.067053521	5.41E-07	0.000333668
Sparc	secreted acidic cysteine rich glycoprotein	0.762553462	1.28E-06	0.000722354
Cd207	CD207 antigen	2.814615455	1.52E-06	0.000834997
Cd300ld4	CD300 molecule like family member D4	3.930944527	4.51E-06	0.002023418
Marco	macrophage receptor with collagenous structure	0.745474916	7.49E-06	0.003144185
Cd209b	CD209b antigen	0.91581455	9.25E-06	0.003723817
Adcy1	adenylate cyclase 1	1.11216131	2.73E-05	0.008828631
Spry4	sprouty RTK signaling antagonist 4	0.807915727	3.01E-05	0.009589767

a Adjusted for multiple comparisons.

## Discussion

RA is a common chronic autoimmune and inflammatory disease, and disease remission remains uncommon with current treatments.^7^ Better understanding of disease pathogenesis is key to the discovery of new and better targets for treatment. To our knowledge, this is the first time that DUSP6 is implicated in the pathogenesis of arthritis severity in a model of RA. DUSP6 KO mice were protected and had lower arthritis severity scores and lower joint histology damage scores and erosions. Additionally, levels of the pathogenic cytokine IL6 were significantly decreased in the blood of DUSP6 KO mice, with increased levels of IL10. T cells and B cells from DUSP6 KO mice also expressed increased levels of IL10, which led us to hypothesize that the DUSP6 KO protection was dependent on IL10. Indeed, the introduction of the IL10 KO into the DUSP6 KO (double KO) abrogated the protection conferred by the DUSP6 KO.

IL10 suppresses immune responses, including reducing complement^8^ and neutrophil activation,^9^ two central components in the pathogenesis of KSIA.^10^^,^^11^ IL10 is produced by different cell types, such as macrophages, T cells, and B cell,^12^^,^^13^^,^^14^ including those infiltrating the RA synovial tissues.^6^ Indeed, we observed increased numbers of IL10-producing CD4^+^ and CD4^−^cells. These included Tr1 and Breg (IL10+) cells, and both may be contributing to the protective effect observed in the present study. Foxp3+ Treg cells can produce IL10; however, we did not observe significant difference in numbers of Foxp3+ Treg cells.

Our findings raise the possibility that DUSP6 has a suppressive effect on the transcription of the IL10 gene. While the direct connection between DUSP6 and IL10 transcription was not examined in this study, Erk is required for the IL10 transcription,^15^ and DUSP6 KO mice have increased Erk activation^16^ and enrichment of Erk pathway genes as described in this study, supporting the model.

DUSP6 is expressed by synovial tissue-infiltrating T cells, B cells, and macrophages,^6^ and it is known to inactivate the MAP kinase signaling pathways, most significantly via de-phosphorylation of Erk.^3^^,^^4^^,^^17^ Either deletion and/or inhibition of DUSP6 increases Erk activation.^3^^,^^4^^,^^17^ Our transcriptomic analyses detected “enrichment of Erk1/Erk2 pathway genes” and “MAP kinase kinase binding” in spleens from DUSP6 KO mice, compared with WT, in line with existing knowledge. Enrichment for “transmembrane RTK activity” was detected, as DUSP6 is known to regulate RTK signaling.^2^^,^^3^ “Wnt-protein binding” and “extracellular matrix” pathways were also enriched.

Among the genes with the most significantly increased expression in DUSP6 KO spleens was IL22ra2. IL22ra2 is an antagonist of IL22, and its levels are down-regulated in the inflamed colon^18^ but up-regulated in human atopic dermatitis and psoriasis lesions in clinical remission^19^ and IL22ra2 has been implicated in suppressing inflammation.^20^ The transcriptional regulation of IL22ra2 has not yet been elucidated, but our observations raise the possibility that DUSP6 may suppress its expression.

Among the down-regulated genes in DUSP6 KO, we observed an enrichment for genes implicated in the regulation of cytokine secretion, suggesting that DUSP6 may regulate the activity of non-RTK receptors also. In support of that, a DUSP6 inhibitor was recently reported to suppress TLR4-induced activation of nuclear factor κB (NF-κB) and the expression of IL1β, IL6, and TNF-α in mouse macrophages.^21^ We observed reduced *in vivo* serum levels of IL6 in arthritic DUSP6 KO mice, further suggesting a role for DUSP6 in the signaling of other receptors.

Neutrophil-mediated immunity and neutrophil-related process were also among the pathways enriched in the down-regulated genes. While we did not observe difference in numbers of neutrophils between DUSP6 KO and WT, neutrophils function can be enhanced by DUSP6, and KO mice were reported to have attenuated acute inflammation after myocardial infarction.^5^ However, that model is not directly translated to ours as it does not involve IL10. But it will be important to examine the role of DUSP6 in neutrophil functions in arthritis.

DUSP6 deletion was also shown to protect mice with dextran sulfate sodium (DSS)-induced colitis, a non-autoimmune model of inflammatory bowel disease,^22^ though the precise mechanism was not clear. DUSP6 KO mice are healthy, further suggesting that *in vivo* inhibition of this phosphatase has potential.

In conclusion, we describe a role for DUSP6 in autoimmune arthritis. To our knowledge, this is the first time that DUSP6 KO mice are studied in a model of autoimmune disease, and we show that these mice are significantly protected and have reduced levels of pro-inflammatory cytokines, and increased levels of IL10 and IL10-producing cells. We further demonstrate a key role for IL10 in mediating the DUSP6 KO protective effect and consider DUSP6 as a potential target for therapeutic development for RA and other autoimmune diseases.

### Limitations of the study

While we examined joint histology, we did not examine the transcriptome of the synovial tissues and how those are affected by the presence or absence of DUSP6. That should be a natural next step.

We described a new role for DUSP6 in the regulation of IL10 expression. It will be important to characterize the underlying mechanism of the DUSP6 regulation on IL10 and IL6 expression, as well as DUSP6-specific target signaling pathways in arthritis.

We also observed an enrichment of neutrophil-related pathways among the down-regulated genes in DUSP6 KO mice. Neutrophils have a central role in RA and KSIA pathogenesis. These observations raise the possibility that DUSP6 may also affect neutrophil migration and/or function in arthritis.

## STAR★Methods

### Key resources table

**Table:** 

REAGENT or RESOURCE	SOURCE	IDENTIFIER
**Antibodies**		

anti-CD4-Vioblue (clone: VIT4)	Miltenyi Biotec, Germany	Cat# 130-114-725
anti-CD25-APC (clone: 4E3)	Miltenyi Biotec, Germany	Cat# 130-113-846
anti-Foxp3-PE (clone: 3G3)	Miltenyi Biotec, Germany	Cat# 130-125-587
anti-CD4-Pacific blue (clone: RM4-5)	Biolegend, San Diego, CA	Cat# 100531
anti-IL-17-PerCP/Cy5.5 (clone: TC11-18H10.1)	Biolegend, San Diego, CA	Cat# 506920
anti-CD45RA-PE (clone: 14.8)	BD Biosciences	Cat# 553380
anti-CD49B-PeCy7 (clone: DX-5)	Biolegend, San Diego, CA	Cat# 108922
anti-LAG3-APC (clone: C9B7W)	BD Biosciences	Cat# 562346
anti-IL10-PerCP/Cy5.5 (clone: JES5-16E3)	Biolegend, San Diego, CA	Cat# 505027
anti-CXCR5-biotin-APC (clone 2G8)	BD Biosciences	Cat# 560615
anti-PD1-PeCy7 (clone: RMP1-30)	Biolegend, San Diego, CA	Cat# 109110
anti-BCL6-PE (Clone: IG191E/A8)	Biolegend, San Diego, CA	Cat# 648304

**Critical commercial assays**		

Milliplex mouse cytokine magnetic bead panel	EMD Millipore, MA.	MCYTMAG-70K-PX32

**Deposited data**		

RNA-sequencing	This study	GSE268216

**Experimental models: Organisms/strains**		

KRN TCR transgenic mice	Mathis/Benoist Lab (Boston, MA)	N/A
DUSP6 KO (Dusp6tm1Jmol)	Jackson Labs, Maine	Strain #009069
IL10 knockout (KO) (B6.129P2-IL10tm1Cgn/J)	Jackson Labs, Maine	Strain #002251
C57BL/6J (wild-type, or WT, which are DUSP6^+/+^ and IL10^+/+^)	Jackson Labs, Maine	Strain #000664
NOD/ShiLtJ	Jackson Labs, Maine	Strain #001976

### Resource availability

#### Lead contact

Further information and requests for resources and reagents should be directed to and will be fulfilled by the lead contact, Percio S. Gulko (percio.gulko@mssm.edu).

#### Materials availability

This study did not generate new unique reagents.

#### Data and code availability

All data reported in this paper will be shared by the lead contact upon request. RNA-seq data have been deposited at NCBI with accession number GSE268216 and are publicly available as of the date of publication. Accession numbers are also listed in the key resources table. This paper does not report original code. Any additional information required to reanalyze the data reported in this paper is available from the lead contact upon request.

### Experimental model and study participant details

#### Mice

DUSP6 KO (Dusp6tm1Jmol), IL-10 knockout (KO) (B6.129P2-IL10tm1Cgn/J), C57BL/6J (wild-type, or WT, which are DUSP6^+/+^ and IL10^+/+^), and NOD/ShiLtJ (NOD) mice were purchased from the Jackson Laboratories (Farmington, CT) and housed under specific pathogen-free conditions. DUSP6/IL10 double KO mice were generated at Mount Sinai by intercrossing the strains twice (F2), following by confirmation of the homozygous KO genotypes and expansion of the strain. Mice 8-12 weeks-old were used in all experiments. KRN TCR transgenic mice (gift from Christophe Benoist, Boston, MA) were crossed with NOD (KRN x NOD F1) to generate the arthritogenic serum (below). Experiments were conducted under a protocol approved by the Mount Sinai Institutional Animal Care and Use Committee and followed the ARRIVE guidelines.

### Method details

#### KRN serum-induced arthritis (KSIA)

Arthritogenic serum was collected from the 60-day-old arthritic offspring of KRN x NOD F1 cross. Serum from different batches was pooled and administered to Male and female DUSP6-KO, DUSP6/IL10 double KO, and WT mice at 100 μL IP on days 0 and 2. Mice typically develop arthritis on day 3, and were scored three times a week for 6 to 15 days (25, 26).

#### Arthritis severity scoring

The clinical arthritis score was determined according to a scoring scale ranging from 0 to 16 per mouse per day as previously reported where 1 = swelling and erythema in a single joint, 2 = swelling and erythema in more than one joint, 3 = swelling of the entire paw and 4 = swelling of paw and inability to bear weight.^1^^,^^23^ Ankle diameters were measure with a digital caliper.

#### Histology and histological scoring

At the end of the arthritis observation period mice were euthanized, the paws fixed in 10% formaldehyde and then decalcified with a solution containing hydrochloric acid and 0.1 M EDTA (Cal-Ex; Fisher Scientific, Fairlawn, NJ). Tissues were embedded in paraffin, and slides prepared and stained with hematoxylin-eosin. The slides were evaluated in a non-blinded manner with a combined scoring system that included the following parameters: synovial inflammation, synovial hyperplasia, cartilage and bone erosions, as previously described^24^^,^^25^^,^^26^^,^^27^^,^^28^ (Methods S1).

#### Cytokine quantification

Twenty-five μL of serum were used to detect cytokine with Milliplex mouse Cytokine magnetic bead panel (EMD Millipore, MA).

#### Flow cytometry analysis

Spleens were collected and individually prepared, stained and analyzed as previously described.^28^ Briefly, splenocyte single cell suspensions (1×10^6^) were stained with fluorescent-labelled monoclonal antibodies for cell surface antigens and incubated for 10 min at 25°C. Cytofix/CytopermTM (BD Bioscience, San Jose, CA) were used to fix and permeabilize cells for intracellular staining. Fixed cells were stained with fluorochrome-conjugated antibodies for 1 h at 4°C in the dark. CD4^+^CD25+Foxp3+ regulatory T cells (Tregs) were stained using anti-CD4-Vioblue (clone: VIT4), anti-CD25-APC (clone: 4E3) and anti-Foxp3-PE (clone: 3G3) (all from Miltenyi, Bergisch Gladbach, Germany). Th17 cells were analyzed using anti-CD4-Pacific blue (clone: RM4-5) and anti-IL-17-PerCP/Cy5.5 (clone: TC11-18H10.1). Tr1 cells were identified by anti-CD4-Pacific blue (clone: RM4-5), anti-CD45RA-PE (clone: 14.8), anti-CD49B-PeCy7 (clone: DX-5), anti-LAG3-APC (clone: C9B7W) and anti-IL-10-PerCP/Cy5.5 (clone: JES5-16E3). Lastly, Tfh cells were stained with anti-CD4-Pacific blue (clone: RM4-5), anti-CXCR5-biotin-APC (clone 2G8), anti-PD1-PeCy7 (clone: RMP1-30), anti-BCL6-PE (Clone: IG191E/A8) and anti-IL 10-PerCP/Cy5.5 (clone: JES5-16E3) (antibodies from Biolegend or BD Bioscience). At least 50,000 cells were acquired per sample. All samples were acquired on a BD LSRII (BD Biosciences) or an Attune (Thermo Fisher) flow cytometer, and analyzed with *De Novo* FCS Express (Pasadena, CA).

#### RNA sequencing

Total RNA isolated extracted from five different spleens from WT and five from DUSP6 KO mice, and quantified by Nanodrop. 400 ng per tissue per mouse was sent to Novogene (Beijing, China) for sequencing and analyses (Methods S2). Volcano plot was the generated with VolcaNoseR.^29^

### Quantification and statistical analysis

Means were compared with the t-test, and medians were compared with the Kruskal-Wallis test (multiple groups) or the Mann-Whitney test (single group comparisons) using GraphPad Prism 10 (San Diego, CA).

## Supplemental information

Document S1, Figures S1, S2, Methods S1, and S2

Table S1. Gene Ontology (GO) pathways and processes enriched among the genes expressed in increased levels in DUSP6 KO spleens

Table S2. Gene Ontology (GO) pathways and processes enriched among the genes expressed in lower levels in DUSP6 KO spleens

Table S3. Differentially expressed genes (DEGs) with increased expression in DUSP6 KO spleens compared with C57BL/6 (WT)

Table S4. Differentially expressed genes (DEGs) with decreased expression in DUSP6 KO spleens compared with C57BL/6 (WT)
